# Topologically distinct intratumoral heterogeneity scores for predicting high-risk pathological grades in invasive lung adenocarcinoma: A multicenter study across four institutions

**DOI:** 10.1371/journal.pdig.0001646

**Published:** 2026-09-08

**Authors:** Shanyue Lin, Yudian Mao, Qian He, Wanyin Qi, Sanhong Zhang, Wei Li, Zhichao Zuo

**Affiliations:** 1 Department of Radiology, Xiangtan Central Hospital(The Affiliated Hospital of Hunan University), Xiangtan, Hunan, China; 2 Department of Radiology, The Affiliated Hospital of Southwest Medical University, Luzhou, Sichuan, China; 3 Department of Radiology, Liuyang Hospital of Traditional Chinese Medicine, Changsha, Hunan, China; 4 Department of Radiology, The Third Xiangya Hospital of Central South University, Changsha, Hunan, China; National Research Council Canada, CANADA

## Abstract

High-risk subtypes of invasive lung adenocarcinoma (IAC), particularly micropapillary- or solid-predominant patterns, are closely associated with poor prognosis. This multicenter retrospective study developed and validated a predictive model for the preoperative identification of these high-risk subtypes using topologically distinct intratumoral heterogeneity (ITH) scores derived from CT images. The study included 1,051 patients with IAC. Two complementary ITH scores were developed: a two-dimensional ITH score, which integrated local radiomics features with global pixel distribution patterns on the largest cross-sectional CT slice, and a three-dimensional ITH score, which extended this quantification across the entire tumor volume. Clinicoradiological features and ITH scores were incorporated as model inputs to construct six base machine learning classifiers and a final stacking ensemble classifier. Model interpretability and robustness were evaluated using SHapley Additive exPlanations (SHAP)-based ablation analyses. An independent dataset from The Cancer Imaging Archive (TCIA) was used for external validation to investigate associations between ITH scores and pathological characteristics, genomic features, recurrence-free survival, and overall survival. The stacking ensemble classifier achieved the best predictive performance, with an area under the receiver operating characteristic curve of 0.875, outperforming models based solely on radiomics features (0.834) or clinicoradiological features (0.792). SHAP analysis identified the 3D ITH score as the most influential contributor to model output, and TCIA validation showed that higher 3D ITH scores were associated with more aggressive tumor biology and poorer survival outcomes. The topologically distinct 3D ITH score may provide a clinically meaningful imaging biomarker for preoperative risk stratification in IAC.

## Author summary

We studied whether patterns hidden inside lung tumors on routine CT scans can help identify patients with more aggressive disease before surgery. Pathologists can determine tumor grade after resection, but treatment decisions often need to be made earlier. We developed a way to measure how fragmented and spatially complex the internal structure of a tumor is, using both a single representative image slice and the full tumor volume. We then combined these measurements with standard clinical and imaging features in a machine learning model. In a multicenter patient cohort, the model using the three-dimensional heterogeneity score performed better than models based only on conventional radiomics or routine clinical imaging features. This score also showed meaningful associations with adverse pathological findings and survival outcomes in an external public dataset. Our findings suggest that spatial tumor heterogeneity on CT images may provide useful preoperative information for risk stratification and individualized surgical planning.

## Introduction

Lung cancer remains the leading cause of cancer-related mortality worldwide, with lung adenocarcinoma constituting the most prevalent histological subtype. The widespread implementation of thin-section computed tomography (CT) screening has markedly increased the detection of early-stage lung adenocarcinoma, which often exhibits substantial histopathological heterogeneity even at an early clinical stage [1,2]. According to the World Health Organization (WHO) classification and refinements proposed by the International Association for the Study of Lung Cancer, invasive lung adenocarcinoma (IAC) typically presents with mixed histological growth patterns [3–5]. Among these, micropapillary- or solid-predominant (MP/S) subtypes are recognized as high-grade patterns, which are strongly associated with aggressive biological behavior, higher recurrence rates, and unfavorable prognosis [6–8]. Surgical resection remains the standard of care for early-stage IAC. However, the optimal surgical extent is dictated by the pathological subtype; specifically, high-grade MP/S-predominant tumors often necessitate standard lobectomy with systematic mediastinal lymph node dissection to minimize recurrence risk, whereas sublobar resection may be sufficient for lower-grade lesions [9,10]. Currently, preoperative identification of these subtypes is challenging. Preoperative biopsy is limited by the small sample volume, which often fails to capture the spatial heterogeneity of the entire tumor, and is associated with risks of complications such as pneumothorax and mediastinal emphysema [11]. Consequently, accurate, noninvasive preoperative assessment of pathological subtypes in IAC is essential for optimizing surgical strategies and prognostic evaluation. Preoperative subtype prediction largely relies on conventional clinicoradiological features derived from CT images, such as tumor size, density, margin characteristics, and morphological signs [12–14]. Although clinically informative, these qualitative features are inherently subjective and susceptible to interobserver variability, limiting their diagnostic precision [15]. Radiomics has emerged as a quantitative imaging approach capable of extracting high-dimensional features that characterize tumor morphology and texture, showing promise in predicting pathological grade and biological behavior [16–19]. Recent multimodal deep-learning work integrating CT images with clinicoradiographic features has also underscored the value of combining image-derived representations with routine clinical descriptors for preoperative IASLC grading [20]. However, conventional radiomics methods typically assume uniform tumor composition when analyzing volumetric regions of interest (ROIs), thereby overlooking critical intratumoral spatial variations and biologically relevant subregions. Furthermore, radiomics models often face challenges regarding limited interpretability and reduced robustness across different imaging protocols and institutions [21–23]. These concerns have also been emphasized in recent end-to-end radiomics reviews, which highlight feature instability, reproducibility, validation bias, domain shift, and clinical translation as key barriers across acquisition, preprocessing, feature engineering, modeling, and evaluation steps [24]. To address these limitations, researchers have turned to topological principles to better quantify intratumoral heterogeneity (ITH). Li et al. [25] proposed a dimensionless “ITH score” that integrates global and local pixel information to quantify ITH. This metric demonstrated superior performance over conventional radiomics in histological subtype classification [26] and the prediction of pathological invasiveness [27–29]. Nevertheless, prior ITH scores were primarily derived from two-dimensional (2D) representations of the tumor’s largest cross-sectional slice, neglecting the full three-dimensional (3D) spatial heterogeneity of the lesion. Moreover, these studies focused predominantly on pure ground-glass nodules (pGGNs) [26,28], limiting their applicability to the broader spectrum of solid and part-solid nodules found in IAC. Recently, Zuo et al. [30] advanced this concept by introducing a “3D ITH score,” calculated from the entire tumor volume, which provides a more comprehensive characterization of volumetric heterogeneity in lung cancer. Building on these findings, the present multicenter study aimed to comprehensively evaluate the clinical value of topologically distinct 2D and 3D ITH scores for the preoperative prediction of MP/S-predominant subtypes in IAC. By integrating clinicoradiological features with these ITH scores, we developed and systematically compared multiple base machine learning models and a stacking ensemble strategy. To ensure transparency and reliability, model interpretability and robustness were explored using SHapley Additive exPlanations (SHAP)-based ablation experiments [31]. Furthermore, the prognostic relevance of these ITH scores was externally validated using data from The Cancer Imaging Archive (TCIA). Our goal was to establish an objective, noninvasive, and clinically applicable imaging biomarker to facilitate preoperative risk stratification and individualized treatment planning for patients with IAC.

## Materials and methods

### Ethics statement

Ethical clearance was obtained from the ethics committees of Xiangtan Central Hospital (No. 2021-07-009), Liuyang Hospital of Traditional Chinese Medicine (No. 2022–016), the Affiliated Hospital of Southwest Medical University (No. KY2020147), and the Third Xiangya Hospital of Central South University (No. Fast-25083). All procedures were conducted in accordance with the Declaration of Helsinki. Because this was a retrospective study using de-identified clinical and imaging data, the requirement for written informed consent was waived by the approving ethics committees.

### Patient population and data acquisition

This multicenter retrospective study was conducted across four medical institutions: Xiangtan Central Hospital, the Affiliated Hospital of Southwest Medical University, Liuyang Hospital of Traditional Chinese Medicine, and the Third Xiangya Hospital of Central South University. We consecutively reviewed medical records of patients with pathologically confirmed IAC who underwent curative intent surgical resection between January 2018 and December 2022. Patients were eligible for inclusion if they met all of the following criteria: (1) postoperative histopathological confirmation of IAC according to the 2021 WHO classification of thoracic tumors; (2) complete surgical resection with systematic lymph node dissection or sampling; (3) availability of preoperative non-contrast-enhanced thin-section chest CT images with a reconstructed slice thickness of ≤1.5 mm, acquired using harmonized imaging protocols [26,28,30]; and (4) detailed pathological reports documenting the proportional composition of histological subtypes, including lepidic, acinar, papillary, micropapillary, and solid patterns. Patients were excluded if they had: (1) received neoadjuvant chemotherapy, radiotherapy, or immunotherapy prior to surgery; (2) CT images with substantial artifacts (e.g., severe respiratory motion or metallic artifacts) that compromised image quality or quantitative analysis; (3) tumors that were indistinguishable from adjacent structures (e.g., due to obstructive pneumonia or atelectasis); or (4) lesions too small for reliable texture analysis (maximum diameter < 5 mm). To assess the generalizability of the proposed model, an independent external validation cohort was derived from The Cancer Imaging Archive (TCIA) public database and screened using identical inclusion and exclusion criteria. A flowchart detailing patient enrollment and cohort selection is provided in S1 Fig. To reduce center-related non-biological variability, all included CT examinations were restricted to non-contrast thin-section chest CT with reconstructed slice thickness of ≤1.5 mm, and all quantitative analyses were performed using a uniform preprocessing and feature-extraction pipeline, including identical lung-window settings, consistent tumor-mask handling, and the same local feature extraction parameters across centers. We did not apply retrospective statistical harmonization such as ComBat in the primary analysis because the study aimed to evaluate model behavior under real-world multicenter acquisition variability and because inappropriate harmonization may remove biologically meaningful between-tumor differences. Instead, robustness was assessed through multicenter training/internal validation and independent TCIA validation, and this issue is explicitly acknowledged as a limitation.

### CT lesion mask segmentation

CT images were exported from the Picture Archiving and Communication System and de-identified. Segmentation was performed using ITK-SNAP software, version 4.0, under standard lung window settings (window width, 1600 HU; window level, −600 HU). A volume of interest (VOI) covering the entire tumor was manually segmented slice-by-slice by a radiologist with 10 years of experience, who was blinded to the specific histopathological subtypes. The segmentation results were subsequently verified by a senior radiologist, and any discrepancies were resolved by consensus. Great care was taken to include both solid and ground-glass components while strictly excluding adjacent normal lung parenchyma, large blood vessels, and chest wall. To enable multidimensional analysis, the slice exhibiting the largest tumor area within the 3D VOI was automatically identified as the representative 2D ROI. These high-quality masks served as the basis for the subsequent extraction of radiomic features and the computation of topologically distinct 2D and 3D ITH scores. To assess segmentation reproducibility, 100 cases were randomly selected and independently re-segmented by a second radiologist who was blinded to histopathological subtype and the original masks. The interobserver reproducibility of the 3D ITH score was quantified using the intraclass correlation coefficient (ICC).

### CT radiological feature evaluation

Radiological features were independently evaluated by two radiologists with more than 10 years of experience, who were blinded to clinical and histopathological information, using standard lung window settings. For each pulmonary nodule, the maximum diameter was measured on the largest axial section, and lobar location was recorded. Attenuation characteristics were classified as pure ground-glass, part-solid, or solid. In addition, morphological features, including margin definition (well-defined vs. ill-defined), lobulation, spiculation, and the vascular convergence sign, were systematically assessed. Discrepancies in qualitative assessments were resolved by consensus through consultation with a third senior radiologist.

### Calculation of topologically distinct intratumoral heterogeneity scores

**Fig 1:**
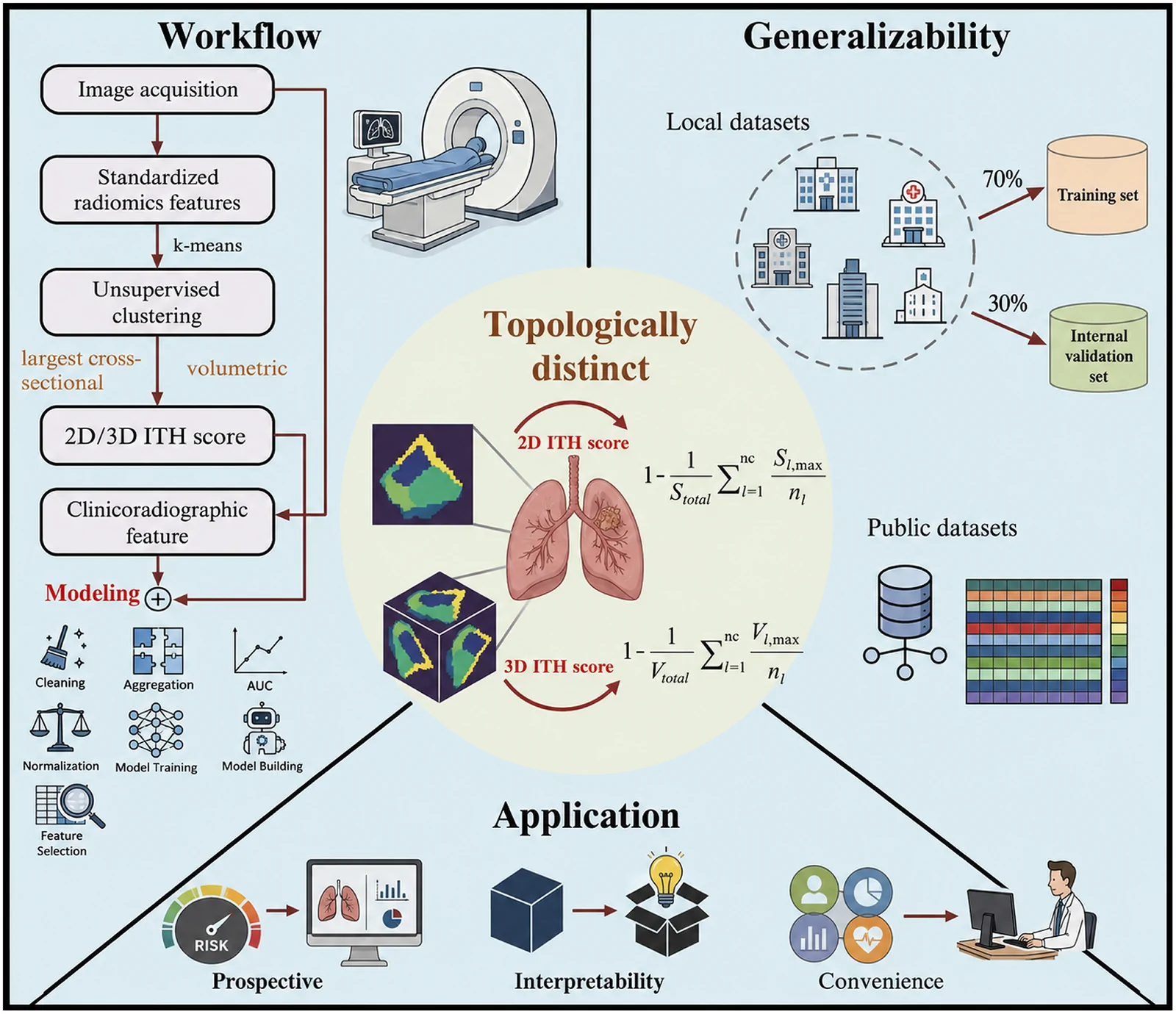
Computational workflow for topologically distinct intratumoral heterogeneity score calculation. The schematic was redrawn by the authors using basic shapes and text only. It summarizes local feature extraction, unsupervised clustering, spatial cluster map generation, topology-aware score derivation, multicenter validation, and clinical application without third-party clip-art or branding.

To quantify the internal heterogeneity of the tumor, we developed a computational framework that transformed radiomic features into topologically distinct tissue subregions. The overall workflow is summarized in Algorithm 1 and schematically illustrated in Fig 1.

**Algorithm 1. Calculation of topologically distinct ITH scores**

Input: CT image I, tumor mask Ω, cluster number K = 6

Output: ITH score (2D or 3D)Step 1: Local feature mapping

1: for each pixel/voxel x ∈ Ω do

2: Extract 104 radiomics feature vector f(x) using sliding window

3: end for

4: Construct feature matrix F = [f(x_1_), …, f(x_n_)]

Step 2: Unsupervised phenotypic clustering

5: Apply k-means clustering on F to obtain K centroids

6: for each pixel/voxel x do

7: Assign cluster label L(x) ∈ {1, …, K} based on minimum Euclidean distance

8: end for

9: Map labels back to spatial coordinates to generate cluster map C

Step 3: Topology-aware quantification

10: Initialize total tumor size S_total_← |Ω|

11: Initialize connectivity accumulator Σ ← 0

12: for each cluster k = 1 to K do

13: Extract binary map C_k_ = {x | L(x) = k}

14: Identify connected components (subregions) in C_k_

15: n_k_ ← count of connected components

16: S_k,max_← size (area or volume) of the largest component

17: Σ ← Σ + (S_k,max_ / n_k_)

18: end for

19: Calculate score:

20: ITH score = 1 − (1 / S_total_) × Σ

21: return ITH score

### Radiomic feature extraction and spatial clustering

As detailed in Step 1 and Step 2 of Algorithm 1, the quantification process began with the generation of a cluster map that delineated discrete tissue subregions based on phenotypic similarities. First, high-dimensional radiomic features, including texture and intensity descriptors, were extracted for each intratumoral pixel (or voxel) using a sliding window approach (kernel size 2 × 2 for 2D; 2 × 2 × 2 for 3D). This resulted in a local feature vector f(x) comprising 104 descriptors for every spatial point within the tumor mask Ω. Subsequently, unsupervised machine learning was employed to group these local features. Using k-means clustering (K = 6) directly on the constructed feature matrix, we partitioned the tumor into distinct phenotypic clusters. Each pixel/voxel was assigned a label corresponding to its nearest cluster centroid. By mapping these labels back to their original spatial coordinates, we constructed a categorical cluster map (C). In this map, spatially contiguous regions sharing the same cluster label represented specific phenotypic subregions, reflecting underlying physiological or metabolic uniformity [30]. The cluster number was selected using an elbow-style internal validation analysis based on the Calinski-Harabasz index across candidate values of K = 2–8. The index reached its maximum at K = 6, supporting this value as the optimal balance between phenotypic granularity and cluster compactness/separation (S2 Fig). To avoid information leakage, cluster-number assessment and downstream model construction were performed within the training workflow before evaluation on the validation cohorts.

### Derivation of the 2D ITH score

The 2D ITH score quantified heterogeneity within the largest axial plane of the nodule. Rather than relying solely on the relative abundance of the generated clusters, the score explicitly incorporated their spatial arrangement and connectivity topology (Step 3 of Algorithm 1).

Specifically, for each cluster type, we identified the number of disconnected subregions and the size of the dominant component. The final 2D ITH score integrated these topological metrics according to Eq (1):


$$\begin{array}{c} \text{2D ITH Score }=\;\text{1}\;-\;\frac{\text{1}}{{\text{S}}_{\text{total}}}\;\times \;\sum_{\text{l}=\text{1}}^{{\text{n}}_{\text{c}}}\frac{{\text{S}}_{\text{l},\text{max}}}{{\text{n}}_{\text{l}}} \end{array}$$ (1)

where nc is the total number of clusters, nl is the count of connected components for cluster l, Sl,max is the area of the largest connected component for that cluster, and Stotal is the total tumor area on the axial slice.

### Derivation of the 3D ITH score

Extending the analysis to the volumetric domain, the 3D ITH score captured anisotropic texture variations and spatial intermixing across the entire tumor volume [30,32,33]. To ensure physiological relevance, the identification of connected components within the 3D cluster map utilized a 26-connectivity topology (encompassing 6 face-, 12 edge-, and 8 corner-adjacent neighbors). This approach preserved the continuity of complex infiltrative patterns that might be fragmented in two-dimensional views. The 3D ITH score was calculated using a volume-weighted dispersion formulation defined in Eq (2):


$$\begin{array}{c} \text{3D ITH Score }=\;\text{1}\;-\;\frac{\text{1}}{{\text{V}}_{\text{total}}}\;\times \;\sum_{\text{l}=\text{1}}^{{\text{n}}_{\text{c}}}\frac{{\text{V}}_{\text{l},\text{max}}}{{\text{m}}_{\text{l}}} \end{array}$$ (2)

where ml represents the count of distinct connected volumes for cluster l, Vl,max denotes the volume of the largest connected component for that cluster, and Vtotal is the total tumor volume.

### Machine learning framework

Six supervised machine learning algorithms—Random Forest (RF), Adaptive Boosting (AdaBoost), Light Gradient Boosting Machine (LightGBM), Gradient Boosting Decision Tree (GBDT), Extreme Gradient Boosting (XGBoost), and Categorical Boosting (CatBoost)—were applied to predict high-grade IAC subtypes. The model inputs consisted of standard clinicoradiological features combined with the calculated topologically distinct ITH scores. Hyperparameters for all classifiers were optimized via grid search within a fivefold cross-validation framework, maximizing the area under the receiver operating characteristic curve (AUC). All preprocessing, feature selection, threshold determination for threshold-dependent metrics, and hyperparameter tuning were confined to the training workflow; the internal validation and TCIA datasets were kept independent for performance assessment to minimize overfitting and data leakage.

As schematically illustrated in S3 Fig, a two-level stacked generalization (stacking) ensemble framework was implemented [29,34–36]. At Level 0, the six base learners generated out-of-fold predictions, which were consolidated into a meta-feature matrix. At Level 1, a logistic regression meta-learner integrated these predictions to produce a final classification of patients into high-risk or low-risk categories.

### Model selection and interpretability analysis

The optimal predictive framework was identified as the classifier achieving the highest AUC on the internal validation set [37]. To clarify the decision-making process and further refine the model by eliminating redundant variables, we implemented a SHAP-guided incremental feature ablation strategy. This procedure, which integrated feature importance ranking with sequential forward selection to determine the most parsimonious feature subset, is formally detailed in Algorithm 2.

**Algorithm 2. SHAP-guided feature selection and ablation**

Input: best trained model M, dataset D, full feature set F = {f_1_, …, f_n_}

Output: optimal feature subset S*

Phase 1: Global importance ranking

1: Compute SHAP values φ_m_(f_i_) for all samples m and features f_i_

2: Calculate global importance score: v̄_i_← (1/M) Σ_m_ |φ_m_(f_i_)|

3: Rank features by importance: F_ranke_d ← sort_v_^-^↓(F) = {f′_1_, f′_2_, …, f′_n_}Phase 2: Incremental ablation study

4: Initialize k* ← 1, AUC_max_ ← 0

5: for k = 1 to N do

6: Construct candidate subset: S_k_ ← {f′_1_, …, f′_k_}

7: Evaluate performance: AUC_k_ ← fivefold CV AUC using S_k_

8: if AUC_k_ > AUC_max_ then

9: AUC_max_← AUC_k_

10: k* ← k

11: end if

12: end for

13: Selection:

14: S* ← S_k_* ▷ Select subset at peak performance

15: return S*

### Comparative experimental design

To validate the stacking strategy, we benchmarked its performance against hard voting (majority rule) and soft voting (probability averaging) using the same base classifiers. Furthermore, to evaluate the incremental diagnostic value of the topologically distinct ITH scores, the full model was compared against two baseline frameworks. The first was a clinicoradiological model relying solely on demographics and semantic CT descriptors (e.g., lobulation, spiculation, and margin status).

The second was a conventional radiomics model constructed from 1,239 initial features. To mitigate overfitting, this radiomics baseline underwent a standard dimensionality reduction pipeline: statistical filtering (Student’s t test or the Mann-Whitney U test, P < 0.05), redundancy removal via Pearson correlation (|r| > 0.9), and feature selection using least absolute shrinkage and selection operator regression [38].

### Statistical analysis

Statistical analyses were performed using Python 3.11.0 and R 4.3.0. Continuous variables were compared using the Student’s t test or the Mann-Whitney U test depending on data normality, while categorical variables were analyzed using the χ2 test. Model performance was evaluated using the AUC, accuracy, sensitivity/recall, precision, and F1-score. For all diagnostic metrics, 95% confidence intervals were calculated using bootstrap resampling. Because AUC is threshold-independent whereas recall, precision, and F1-score are threshold-dependent, the revised performance tables report threshold-dependent metrics using the optimal diagnostic threshold derived from receiver operating characteristic analysis. This was done to better reflect the clinically relevant balance between identifying MP/S-predominant tumors and avoiding excessive false-positive predictions. Decision curve analysis (DCA) was conducted to assess clinical net benefit. Survival outcomes were estimated using the Kaplan-Meier method and compared via the log-rank test. Correlations between ITH scores and biological features were assessed using Spearman’s rank correlation coefficients. A two-sided P value < 0.05 was considered statistically significant.

## Results

### Patient characteristics

This multicenter study included a total of 1,051 patients with pathologically confirmed IAC collected across four institutions. Among them, 206 patients (19.6%) exhibited high-risk histological subtypes, defined as MP/S patterns, while the remaining 845 patients (80.4%) were classified as low-risk subtypes. The cohort was randomly divided into a training set (n = 735) and an internal validation set (n = 316). The proportion of high-risk cases was well balanced between the two cohorts, comprising 19.6% (144/735) of the training set and 19.6% (62/316) of the internal validation set. As summarized in Table 1, baseline clinicoradiological characteristics were generally balanced between the training and internal validation cohorts, although age differed statistically between cohorts (P = 0.020).

**Table 1:** Comparison of clinicoradiological features between the training and internal validation sets.

Variables	Total (n = 1051)	Training set (n = 735)	Validation set (n = 316)	P value
Pathological type, n (%)				1.000
Non-MP/S-predominant	845 (80.4)	591 (80.4)	254 (80.4)	
MP/S-predominant	206 (19.6)	144 (19.6)	62 (19.6)	
Sex, n (%)				0.767
Female	654 (62.2)	460 (62.6)	194 (61.4)	
Male	397 (37.8)	275 (37.4)	122 (38.6)	
Age (y), median (Q1, Q3)	59 (53, 67)	59 (52, 66)	61 (53, 68)	0.020
Nodule size (mm), median (Q1, Q3)	21.6 (16.4, 27.3)	21.6 (16.2, 27.2)	21.7 (16.4, 27.4)	0.234
CT density, n (%)				0.459
pGGN	339 (32.3)	242 (32.9)	97 (30.7)	
Part-solid	339 (32.3)	241 (32.8)	98 (31.0)	
Solid	373 (35.5)	252 (34.3)	121 (38.3)	
Location, n (%)				0.162
RUL	374 (35.6)	249 (33.9)	125 (39.6)	
RLL	74 (7.0)	48 (6.5)	26 (8.2)	
RML	190 (18.1)	132 (18.0)	58 (18.4)	
LUL	270 (25.7)	198 (26.9)	72 (22.8)	
LLL	143 (13.6)	108 (14.7)	35 (11.1)	
Boundary, n (%)				0.688
Well-defined	749 (71.3)	527 (71.7)	222 (70.3)	
Ill-defined	302 (28.7)	208 (28.3)	94 (29.7)	
Lobulation sign, n (%)				0.452
Absent	350 (33.3)	239 (32.5)	111 (35.1)	
Present	701 (66.7)	496 (67.5)	205 (64.9)	
Spiculation sign, n (%)				0.586
Absent	444 (42.2)	306 (41.6)	138 (43.7)	
Present	607 (57.8)	429 (58.4)	178 (56.3)	
Vascular convergence sign, n (%)				0.224
Absent	220 (20.9)	146 (19.9)	74 (23.4)	
Present	831 (79.1)	589 (80.1)	242 (76.6)	
Vacuole sign, n (%)				0.941
Absent	828 (78.8)	580 (78.9)	248 (78.5)	
Present	223 (21.2)	155 (21.1)	68 (21.5)	
Pleural indentation sign, n (%)				0.751
Absent	327 (31.1)	226 (30.7)	101 (32.0)	
Present	724 (68.9)	509 (69.3)	215 (68.0)	
Shape, n (%)				0.703
Regular	440 (41.9)	311 (42.3)	129 (40.8)	
Irregular	611 (58.1)	424 (57.7)	187 (59.2)	
2D ITH score, median (Q1, Q3)	0.5 (0.4, 0.7)	0.5 (0.4, 0.7)	0.6 (0.4, 0.7)	0.554
3D ITH score, median (Q1, Q3)	0.9 (0.7, 0.9)	0.9 (0.7, 0.9)	0.9 (0.7, 0.9)	0.108

Abbreviations: MP/S, micropapillary/solid; pGGN, pure ground-glass nodule; RUL, right upper lobe; RLL, right lower lobe; RML, right middle lobe; LUL, left upper lobe; LLL, left lower lobe; ITH, intratumoral heterogeneity.

For external validation, an independent cohort of 113 patients was obtained from TCIA. Within this external dataset, 22 patients (19.5%) were identified as having high-risk pathological subtypes, demonstrating a distribution consistent with that of the internal cohorts.

### Robustness analyses for cluster-number selection and segmentation reproducibility

The Calinski-Harabasz index increased from K = 2 to K = 6 and declined thereafter, supporting K = 6 as the optimal clustering granularity for the topology-aware ITH framework (S2 Fig). This result provided an empirical rationale for the fixed cluster number used in the main analysis. In addition, the interobserver reproducibility analysis performed in 100 randomly selected cases demonstrated excellent agreement between the two radiologists for the 3D ITH score (ICC = 0.916), supporting the robustness of the proposed metric to manual segmentation variability.

### Performance comparison with base classifiers

After recalculating threshold-dependent metrics using the optimal diagnostic threshold, the stacking ensemble classifier achieved the highest AUC (0.875, 95% CI: 0.830–0.920), F1-score (0.588, 95% CI: 0.468–0.708), precision (0.592, 95% CI: 0.469–0.715), and recall/sensitivity (0.584, 95% CI: 0.461–0.707), with an accuracy of 0.816 (95% CI: 0.773–0.859) among all models (Table 2). These results indicate that the revised threshold substantially improved the balance between sensitivity and precision while retaining the threshold-independent discriminative advantage of the stacking model. Decision curve analysis indicated that the stacking ensemble provided the highest net clinical benefit across the majority of threshold probabilities (S4 Fig).

**Table 2:** Comparison of diagnostic performance between base machine learning models and the stacking ensemble classifier.

Models	Accuracy (95% CI)	AUC (95% CI)	F1-score (95% CI)	Precision (95% CI)	Recall (95% CI)
Stacking ensemble	0.816 (0.773-0.859)	0.875 (0.830-0.920)	0.588 (0.468-0.708)	0.592 (0.469-0.715)	0.584 (0.461-0.707)
RF	0.808 (0.765-0.851)	0.845 (0.798-0.892)	0.538 (0.415-0.661)	0.542 (0.418-0.666)	0.535 (0.411-0.659)
XGBoost	0.805 (0.761-0.849)	0.794 (0.743-0.845)	0.515 (0.391-0.639)	0.518 (0.393-0.643)	0.512 (0.388-0.636)
LightGBM	0.812 (0.769-0.855)	0.828 (0.779-0.877)	0.551 (0.428-0.674)	0.554 (0.430-0.678)	0.548 (0.424-0.672)
GBDT	0.806 (0.762-0.850)	0.834 (0.786-0.882)	0.528 (0.405-0.651)	0.531 (0.407-0.655)	0.525 (0.401-0.649)
AdaBoost	0.810 (0.767-0.853)	0.852 (0.806-0.898)	0.555 (0.432-0.678)	0.558 (0.434-0.682)	0.552 (0.428-0.676)
CatBoost	0.814 (0.771-0.857)	0.865 (0.819-0.911)	0.575 (0.454-0.696)	0.578 (0.455-0.701)	0.572 (0.449-0.695)

Abbreviations: RF, random forest; XGBoost, extreme gradient boosting; LightGBM, light gradient boosting machine; GBDT, gradient boosting decision tree; AdaBoost, adaptive boosting; AUC, area under the receiver operating characteristic curve; CI, confidence interval. Threshold-dependent metrics were calculated using the optimal diagnostic threshold, and 95% CIs were estimated using bootstrap resampling.

### Comparative experiments

We performed a two-stage comparative analysis to validate the proposed framework. First, we evaluated different ensemble strategies (Table 3). The stacking strategy achieved the highest diagnostic performance (AUC = 0.875, 95% CI: 0.830–0.920; F1-score = 0.588, 95% CI: 0.468–0.708; recall = 0.584, 95% CI: 0.461–0.707, at the optimal diagnostic threshold), substantially outperforming hard voting (AUC = 0.689, 95% CI: 0.631–0.747) and moderately surpassing soft voting (AUC = 0.850, 95% CI: 0.804–0.896), indicating superior predictive stability and a more clinically balanced sensitivity–precision profile.

**Table 3:** Comparison of diagnostic performance among ensemble strategies.

Models	Accuracy (95% CI)	AUC (95% CI)	F1-score (95% CI)	Precision (95% CI)	Recall (95% CI)
Hard voting	0.835 (0.794-0.876)	0.689 (0.631-0.747)	0.435 (0.320-0.550)	0.667 (0.505-0.829)	0.323 (0.207-0.439)
Soft voting	0.839 (0.798-0.880)	0.850 (0.804-0.896)	0.495 (0.375-0.615)	0.641 (0.490-0.792)	0.403 (0.281-0.525)
Stacking ensemble	0.816 (0.773-0.859)	0.875 (0.830-0.920)	0.588 (0.468-0.708)	0.592 (0.469-0.715)	0.584 (0.461-0.707)

Abbreviations: AUC, area under the receiver operating characteristic curve; CI, confidence interval. Threshold-dependent metrics were calculated using the optimal diagnostic threshold, and 95% CIs were estimated using bootstrap resampling.

Second, we benchmarked the full model (integrating ITH scores) against baseline models (Table 4). The full model outperformed the clinicoradiological model (AUC, 0.875 [95% CI: 0.830–0.920] vs. 0.792 [95% CI: 0.741–0.843]) and the conventional radiomics model (AUC = 0.834, 95% CI: 0.786–0.882), and the use of the optimal diagnostic threshold yielded a substantially improved F1-score of 0.588 (95% CI: 0.468–0.708) for the full model. ROC analysis (S5 Figa) confirmed the full model achieved the largest AUC, while DCA (S5 Figb) demonstrated it offered the highest net clinical benefit across most threshold probabilities compared with alternative models.

**Table 4:** Comparison of diagnostic performance among the radiomics signature, clinicoradiological model, and stacking ensemble classifier.

Models	Accuracy (95% CI)	AUC (95% CI)	F1-score (95% CI)	Precision (95% CI)	Recall (95% CI)
Radiomics signature	0.839 (0.798-0.880)	0.834 (0.786-0.882)	0.400 (0.289-0.511)	0.976 (0.880-1.000)	0.274 (0.163-0.385)
Clinicoradiological	0.797 (0.753-0.841)	0.792 (0.741-0.843)	0.135 (0.052-0.218)	0.972 (0.855-1.000)	0.081 (0.013-0.149)
Stacking ensemble	0.816 (0.773-0.859)	0.875 (0.830-0.920)	0.588 (0.468-0.708)	0.592 (0.469-0.715)	0.584 (0.461-0.707)

Abbreviations: AUC, area under the receiver operating characteristic curve; CI, confidence interval. Threshold-dependent metrics were calculated using the optimal diagnostic threshold, and 95% CIs were estimated using bootstrap resampling.

### Model interpretability and ablation analysis

**Fig 2:**
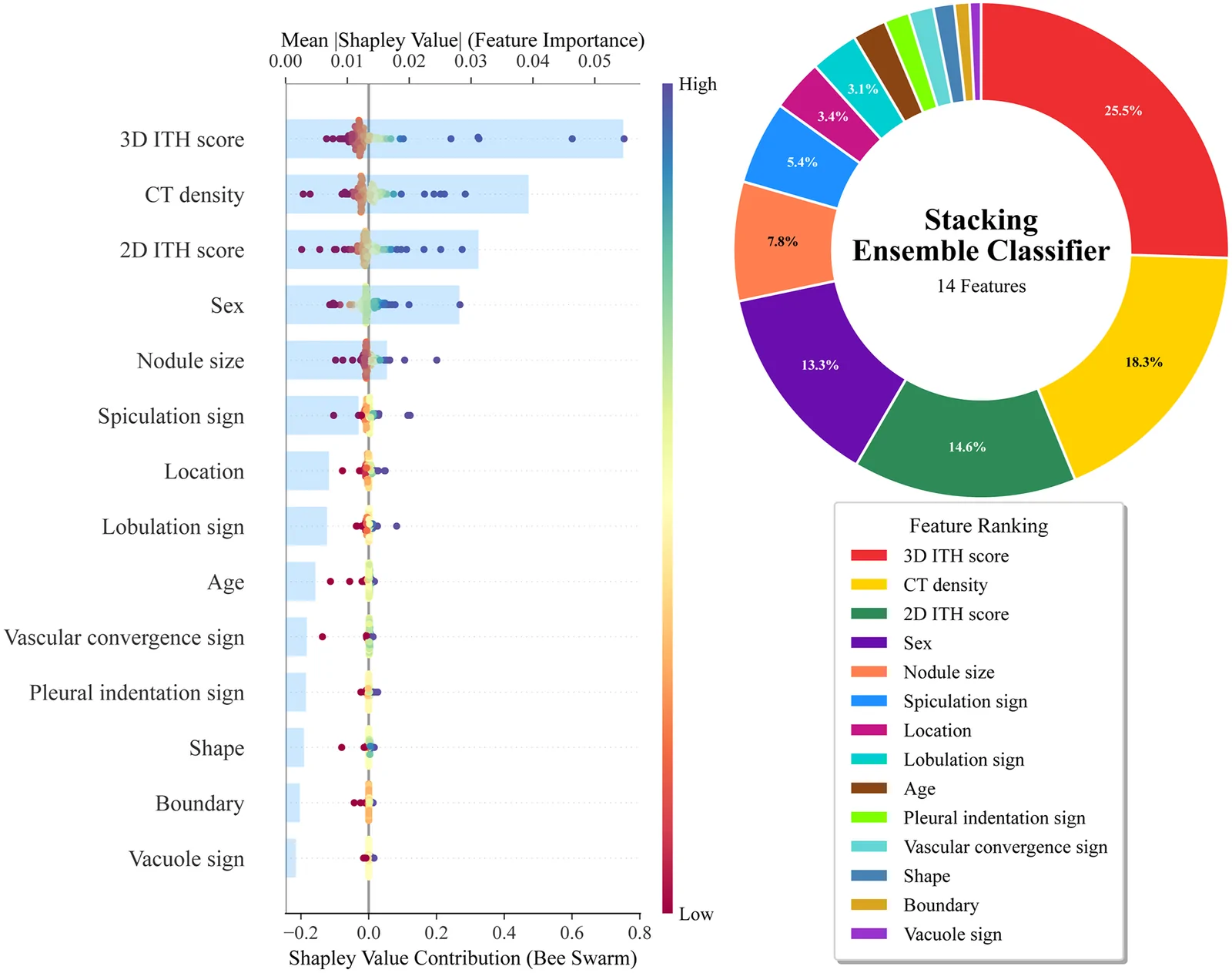
Global feature importance. The 3D ITH score accounts for the highest proportion of feature contribution, followed by CT density and the 2D ITH score.

SHAP analysis of the stacking ensemble revealed that the CatBoost classifier contributed most significantly to the meta-learner’s decision-making process (S6 Fig). Regarding global feature importance, the 3D ITH score was identified as the dominant predictor, accounting for approximately 25.5% of the model’s decision weight (Fig 2). Higher 3D ITH values were consistently associated with positive SHAP contributions, indicating a higher probability of high-risk MP/S subtypes.

**Fig 3:**
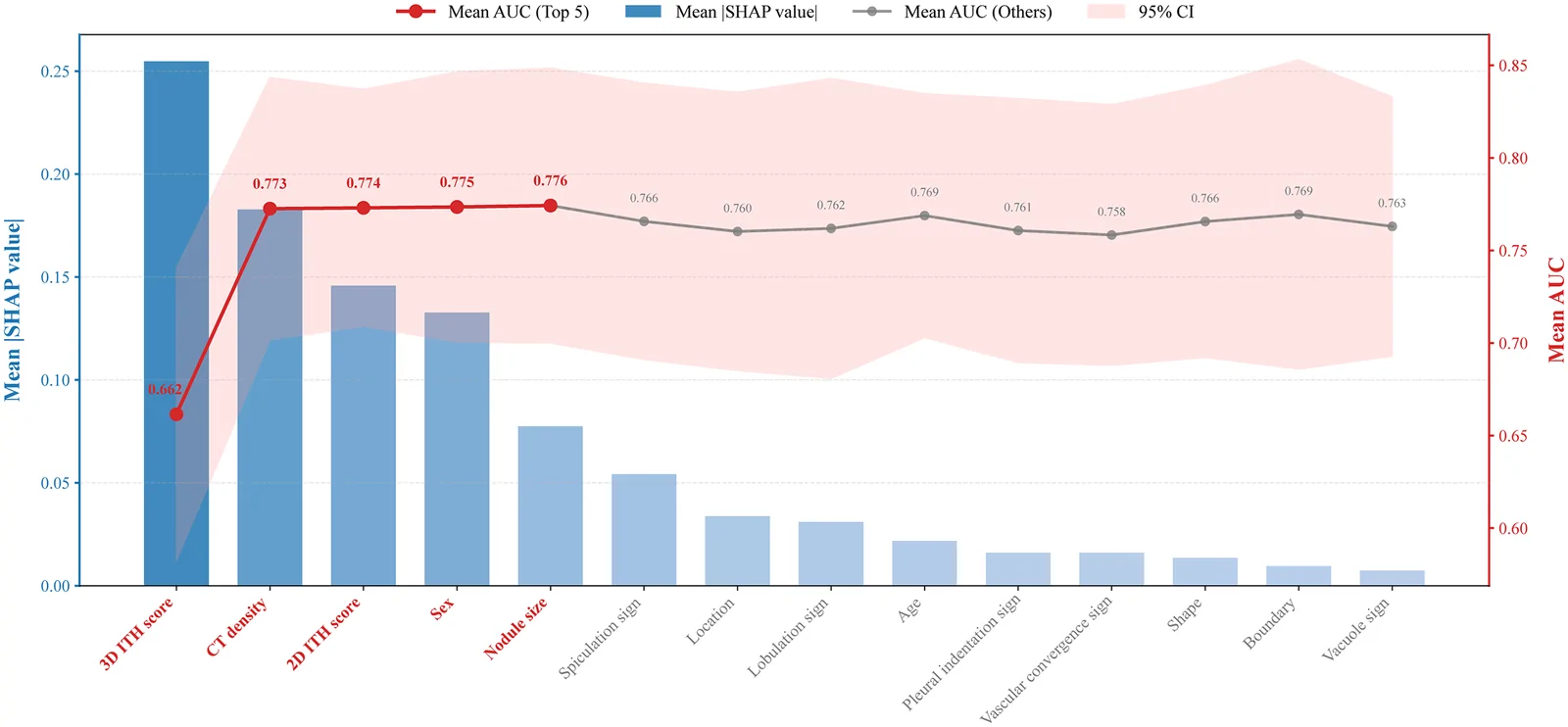
Incremental ablation analysis. The curve shows that model performance plateaued after incorporating the top five features.

The incremental ablation study demonstrated that model performance improved rapidly with the addition of the top-ranked features (Fig 3). Diagnostic discriminative power plateaued after the inclusion of the top five variables: 3D ITH score, CT density, 2D ITH score, sex, and nodule size. This finding confirms that robust classification can be achieved using a parsimonious feature subset.

### Clinicopathological associations and prognostic stratification of the 3D ITH score

**Fig 4:**
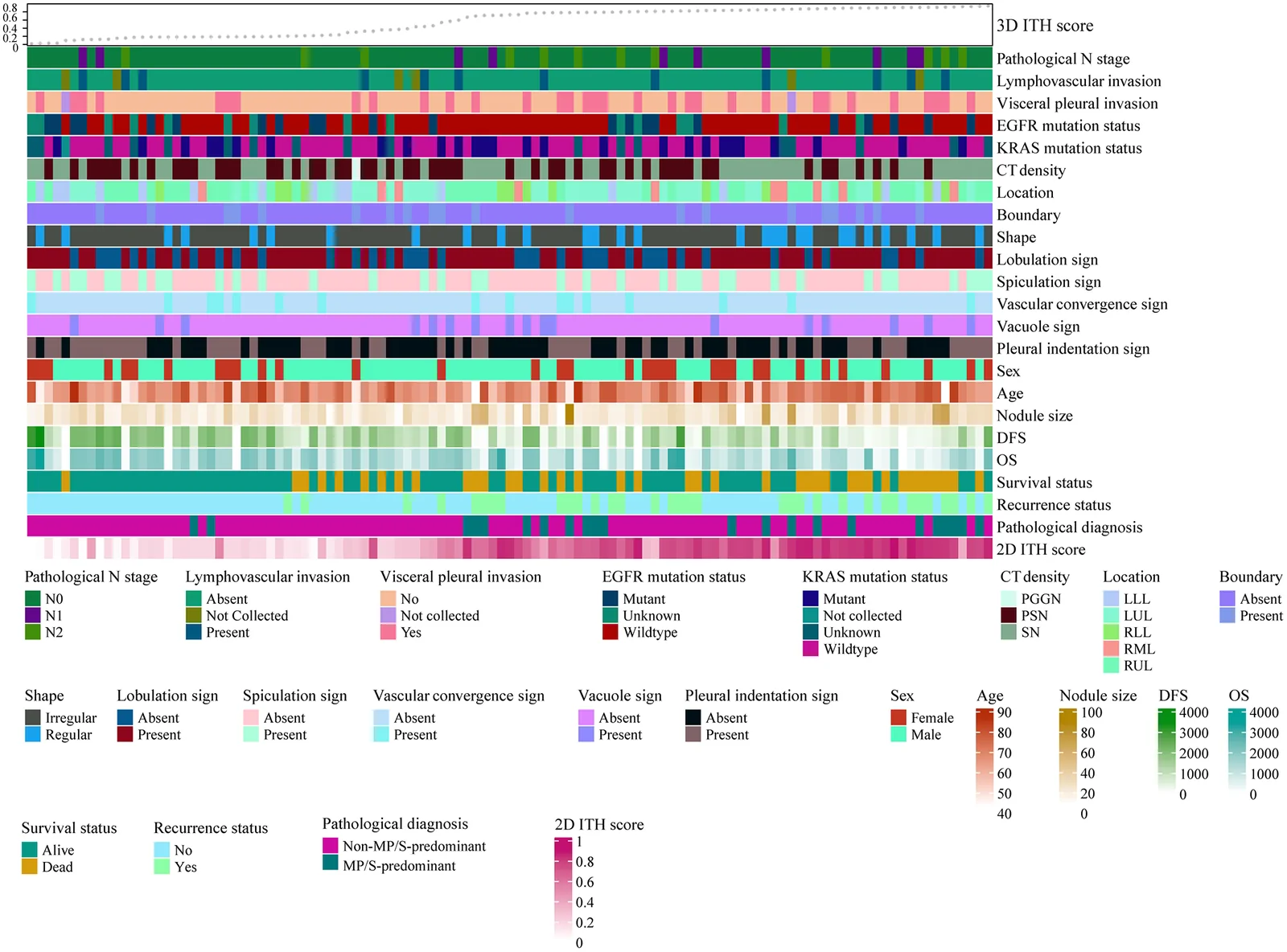
Clinicopathological correlations. Heatmap showing positive associations between the 3D ITH score and adverse pathological variables.

In the external TCIA validation cohort, the correlation heatmap (Fig 4) revealed that the 3D ITH score was positively associated with aggressive pathological markers, including advanced N stage, lymphovascular invasion, and pleural invasion.

**Fig 5:**
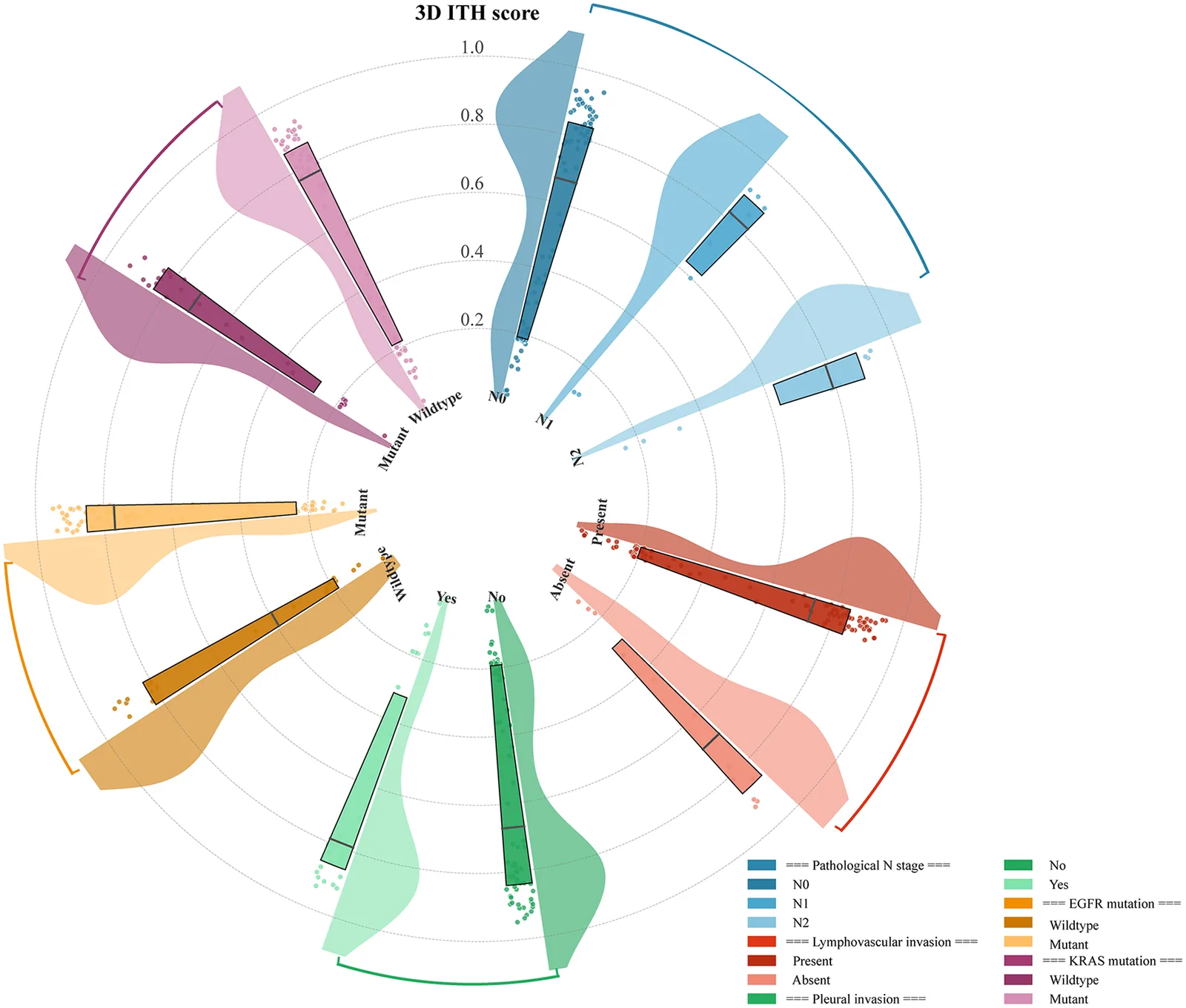
Distribution analysis. Raincloud plots showing higher 3D ITH scores in patients with adverse clinical outcomes.

Furthermore, quantitative distribution analysis (Fig 5) demonstrated that 3D ITH scores were significantly elevated in patients harboring epidermal growth factor receptor (EGFR) mutations, as well as in those who experienced tumor recurrence or mortality.

**Fig 6:**
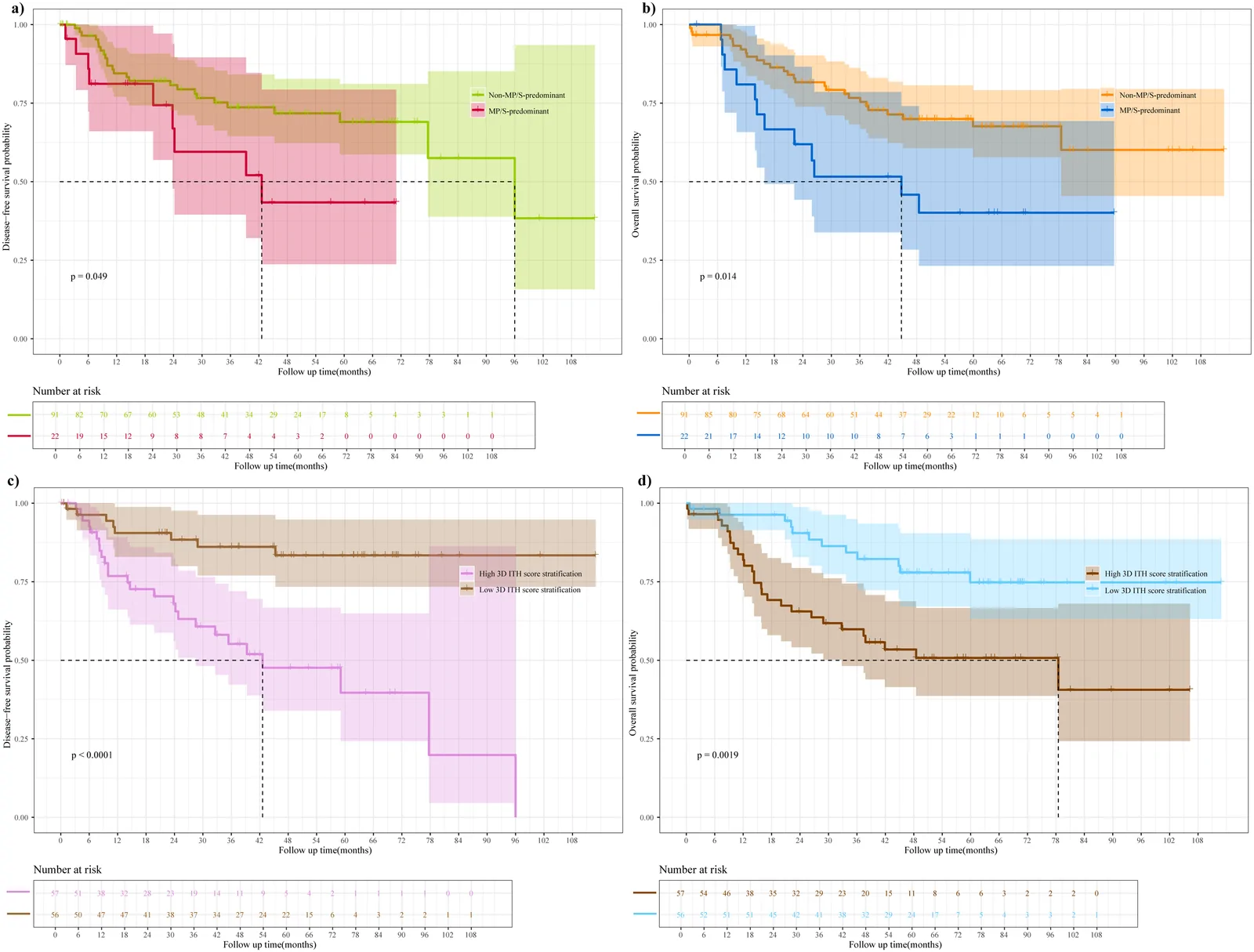
Survival analysis. Kaplan-Meier curves for overall survival and recurrence-free survival stratified by high and low 3D ITH scores.

Kaplan-Meier survival analyses further confirmed the prognostic value of the metric. Patients in the high-risk group (stratified by median 3D ITH score) exhibited significantly poorer overall survival (OS) and recurrence-free survival (RFS) compared with the low-risk group (log-rank P < 0.05; Fig 6).

## Discussion

This multicenter study demonstrated that a stacking ensemble learning framework, integrating topologically distinct ITH scores with clinicoradiological features, robustly predicted high-risk MP/S subtypes in IAC. The proposed model achieved superior diagnostic performance (AUC = 0.875) compared with traditional radiomics signatures and single machine learning classifiers. After applying the optimal diagnostic threshold, the model also showed a more balanced threshold-dependent profile, with recall/sensitivity increasing to 0.584 and F1-score increasing to 0.588. This refinement is clinically relevant because preoperative identification of MP/S-predominant tumors requires a reasonable balance between avoiding missed high-risk cases and preventing unnecessary overtreatment. Notably, the 3D ITH score, which quantified volumetric pixel distribution and texture complexity, emerged as the dominant predictive feature, highlighting the value of topological metrics in decoding tumor heterogeneity. Preoperative identification of high-risk MP/S subtypes is critical for optimizing surgical decision-making. Although recent phase 3 trials (JCOG0802, CALGB140503) have established sublobar resection as a standard for small peripheral non-small cell lung cancers, tumors harboring aggressive MP/S components are associated with a significantly increased risk of locoregional recurrence. Consequently, sublobar resection may be oncologically insufficient for these high-grade lesions [39,40]. Our predictive model serves as a vital decision-support tool, potentially identifying patients who would benefit from standard lobectomy with systematic lymph node dissection. Furthermore, as the presence of MP/S components increasingly influences adjuvant treatment strategies, high-risk patients identified by our model could be candidates for more rigorous surveillance or adjuvant chemotherapy [9,10].

We addressed the limitations of conventional imaging by moving beyond standard radiomics to quantify topological heterogeneity. While tumor heterogeneity is a hallmark of malignancy [41], traditional radiomics often averages features across the region of interest, potentially obscuring localized variations [21]. Although Li et al. introduced a 2D ITH score based on topological principles [25], 2D analysis inherently neglects anisotropic tumor growth in the z-axis. Our study advanced this methodology by implementing a 3D ITH score that extended topological clustering to the entire voxel grid. This volumetric approach preserved the spatial continuity of tumor habitats, providing a more comprehensive characterization of the complex internal architecture inherent to high-grade histologic subtypes [30]. Conceptually, this strategy is consistent with phenotype-centric representation frameworks recently discussed in histomics, in which raw images are transformed into structured descriptors of morphology, texture, spatial organization, and architectural context rather than being treated only as latent black-box inputs [42]. Although our work is based on CT rather than histology images, the clustered radiomic subregions can be interpreted as imaging phenotypes or tumor habitats that summarize local differences in attenuation, texture, and spatial organization. Technically, the stacking ensemble strategy demonstrated superior stability and accuracy compared with individual base classifiers. The hierarchical structure of the meta-learner effectively integrated complementary decision boundaries, reducing variance and mitigating overfitting [34]. Beyond predictive performance, the SHAP-based ablation analysis confirmed the robustness of our feature selection. The 3D ITH score consistently ranked as the top contributor to model output. Notably, diagnostic performance plateaued after the inclusion of only the top five features. This finding challenges the “more is better” assumption often seen in high-dimensional radiomics, suggesting that a parsimonious model driven by biologically relevant heterogeneity metrics is sufficient for high-precision prediction. A distinct strength of this study was the external validation on the TCIA dataset, which extended our evaluation from diagnostic accuracy to biological relevance. We observed a significant positive correlation between elevated 3D ITH scores and aggressive clinicopathological features, including advanced T/N stages and lymphovascular invasion. Furthermore, survival analysis revealed that patients with high 3D ITH scores experienced significantly shorter RFS and OS. Biologically, a higher 3D ITH score indicates that intratumoral phenotypic clusters are more fragmented, spatially intermixed, and less dominated by a single connected component. Such a pattern may correspond to mixed histological growth patterns, heterogeneous cellular density, stromal reaction, necrotic or fibrotic foci, and uneven invasive fronts. Therefore, the 3D ITH score should be interpreted as a CT-derived spatial phenotype associated with aggressive tumor architecture rather than as direct evidence of a specific molecular mechanism. These results align with the hypothesis that high imaging heterogeneity may serve as an imaging surrogate for underlying tumor evolution and phenotypic diversity, which contribute to tumor progression and therapeutic resistance [41]. Our study has limitations. First, despite the multicenter design, the retrospective nature of data collection may introduce selection bias. Prospective validation is required to confirm clinical utility. Second, although all CT examinations met predefined thin-section acquisition criteria and were processed using the same computational pipeline, residual multicenter imaging variability related to scanner vendor, reconstruction kernel, and acquisition parameters could not be completely eliminated. We intentionally did not apply statistical harmonization in the primary analysis to preserve evaluation under real-world imaging variability, but future studies should compare harmonized and non-harmonized pipelines. Third, although an interobserver reproducibility analysis in 100 randomly selected cases showed excellent agreement for the 3D ITH score (ICC = 0.916), ITH score calculation still relies on manual segmentation and remains labor-intensive; integration with fully automated deep learning segmentation could facilitate clinical workflow implementation. Fourth, the TCIA external validation cohort was relatively small, particularly for survival and genomic subgroup analyses; therefore, these analyses should be considered exploratory and require confirmation in larger external cohorts with multivariable survival modeling. Finally, the direct biological underpinnings connecting topological ITH scores to specific molecular pathways (e.g., EGFR, KRAS mutations) require further elucidation through radiogenomic spatial transcriptomics.

## Conclusion

The topologically distinct 3D ITH score represents a robust and biologically relevant imaging biomarker for the preoperative identification of high-risk pathological subtypes in IAC. The proposed stacking ensemble model offers a precise, noninvasive tool for risk stratification, potentially guiding individualized surgical strategies and adjuvant treatment planning in the era of precision medicine.

## Supporting information

S1 Fig Patient enrollment and cohort selection. Flowchart showing patient screening, exclusions, and cohort allocation.
(TIF)

S2 Fig Cluster‑number selection for k‑means phenotypic clustering. The Calinski‑Harabasz index was evaluated across candidate cluster numbers. The maximum value was observed at K = 6, supporting the selected clustering granularity.
(TIF)

S3 Fig Two‑level stacking ensemble framework. The schematic was redrawn by the authors and all software logos or branding were removed. Six base classifiers generate out‑of‑fold predictions that are integrated by a logistic regression meta‑learner.
(TIF)

S4 Fig Diagnostic performance of the machine learning models. (A) Receiver operating characteristic curves show that the stacking ensemble classifier achieved the highest area under the receiver operating characteristic curve of 0.875. (B) Decision curve analysis shows that the stacking ensemble model provided the highest net benefit across most threshold probabilities compared with the other classifiers and the treat‑all or treat‑none strategies.
(TIF)

S5 Fig Comparative diagnostic performance. (A) Receiver operating characteristic curves for the radiomics signature, clinicoradiological model, and stacking ensemble model. (B) Decision curve analysis showing the net clinical benefit of the models. The stacking ensemble model achieved the highest area under the receiver operating characteristic curve of 0.875 compared with the radiomics signature and clinicoradiological model.
(TIF)

S6 Fig Meta‑learner decision analysis. SHAP values quantify the relative contribution of each base classifier to the meta‑learner’s final prediction.
(TIF)
