# Supplementary material for: Topologically distinct intratumoral heterogeneity scores for predicting high-risk pathological grades in invasive lung adenocarcinoma: A multicenter study across four institutions
Source: PLOS Digit Health. 2026 Sep 8;5(9):e0001646. doi: 10.1371/journal.pdig.0001646 (PMC13552742; doi:10.1371/journal.pdig.0001646)
Supplement: Supplementary file 1 [file pdig.0001646.s007.pdf]

# Response to Reviewers

## Topologically distinct intratumoral heterogeneity scores for predicting high-risk pathological grades in invasive lung adenocarcinoma: A multicenter study across four institutions

Shanyue Lin, Qian He, Yudian Mao, Wanyin Qi, Sanhong Zhang, Wei Li*, Zhichao Zuo*

June 5, 2026

Dear Editors and Reviewers,

We sincerely thank the editors and reviewers for their constructive comments and careful evaluation of our manuscript. We have revised the manuscript point by point. All revisions are highlighted in red in the marked manuscript, and a clean version is also provided. Below, we reproduce each editorial/reviewer comment first and then provide our point-by-point response and the corresponding changes made in the revised manuscript.

## Editorial and journal requirements

### Editorial requirement 1. Data availability

> **Reviewer/editor comment:** In this instance it seems there may be acceptable restrictions in place that prevent the public sharing of your minimal data. However, in line with our goal of ensuring long-term data availability to all interested researchers, PLOS' Data Policy states that authors cannot be the sole named individuals responsible for ensuring data access. Before we proceed with your manuscript, please also provide non-author contact information (phone/email/hyperlink) for a data access committee, ethics committee, or other institutional body to which data requests may be sent. Please also provide details on how you will ensure persistent or long-term data storage and availability.

> **Response:** We agree with the editorial requirement. We revised the Data Availability statement to avoid reliance on the corresponding author as the sole data access contact. Following the format used in our previous PLOS Digital Health publication, data requests should be submitted as a detailed research proposal to a non-author institutional route: the Ethics Committee/Data Access Committee at the Third Xiangya Hospital of Central South University (dbxy3yy@163.com). We also clarified that raw CT images and patient-level clinical records cannot be publicly posted because they may contain potentially identifiable human participant information, but de-identified derived data and analysis code required to reproduce the main findings are available upon institutional review. Long-term storage is maintained on secure institutional research data servers with routine backup according to hospital data governance requirements.

**Changes made in the revised manuscript:** The Data Availability section was revised to read: "Because the underlying CT images and patient-level clinical records may contain potentially identifiable human participant information, the raw images and raw clinical records cannot be posted in a public repository. All relevant de-identified data underlying the findings of this study, including the derived feature matrix and analysis code required to reproduce the main results, are available upon institutional review and can be accessed by submitting a detailed research proposal to the Ethics Committee/Data Access Committee at the Third Xiangya Hospital of Central South University (dbxy3yy@163.com). Requests will be reviewed in accordance with institutional regulations, ethics approval requirements, and applicable privacy laws. Long-term storage of the study data is maintained on secure institutional research data servers with routine backup according to hospital data governance requirements."

### Editorial requirement 2. Copyright, logos, and figure compliance

**Reviewer/editor comment:** Some material included in your submission may be copyrighted. Potential copyright issues: Figures 2, 3: Please confirm whether you drew the images/clip-art within the figure panels by hand. If you did not draw the images, please provide a link to the source of the images or icons and their license/terms of use, or written permission from the copyright holder to publish the images or icons under our CC-BY 4.0 license. Figure 3 contains branding/a logo. We are not permitted to publish this under our CC-BY 4.0 license, even with permission. We ask that you please remove or replace it.

**Response:** We agree and have removed all potentially copyrighted elements. The revised schematic figures contain no third-party clip-art, TCIA logo, software branding, or product logos. The workflow figure was redrawn using author-generated basic shapes and text only. The two-level stacking framework was also redrawn without LightGBM, XGBoost, CatBoost, or other software logos. These changes ensure compatibility with the CC-BY 4.0 publication license used by PLOS.

**Changes made in the revised manuscript:** Figures were replaced or reorganized. The computational workflow figure and stacking framework were regenerated as author-drawn schematic diagrams without third-party icons or branding. Selected non-essential figures were moved to Supporting information and renumbered as S1–S6 Figs. Separate figure files will be uploaded according to PLOS requirements.

### Editorial requirement 3. Ethics Statement

**Reviewer/editor comment:** Please ensure that your Ethics Statement is available in its entirety at the beginning of your Methods section, under a subheading "Ethics Statement".

**Response:** We agree. The complete ethics information has been moved to the beginning of the Materials and methods section under the exact subheading "Ethics Statement", as requested by PLOS.

**Changes made in the revised manuscript:** The revised manuscript now includes a complete Ethics Statement at the beginning of the Materials and methods section.

### Editorial requirement 4. Manuscript source file and separate figures

**Reviewer/editor comment:** We ask that a manuscript source file is provided at Revision. Please upload your manuscript file as a .doc, .docx, .rtf or .tex. Please upload separate figure files in .tif or .eps format. Also, remove the figures from your manuscript file but keep the legends.

**Response:** We have prepared the revised manuscript as a .tex source file and will upload it at revision. We will also upload separate figure files according to the journal's figure requirements. In the manuscript source, figure legends have been retained while figures can be provided as separate files.

**Changes made in the revised manuscript:** A revised .tex manuscript source file, clean manuscript, marked manuscript, supporting information legends, and separate figure files have been prepared for submission.

# Reviewer #1

## Reviewer #1 comment

> **Reviewer/editor comment:** The manuscript presents a well-structured multicenter study proposing topologically derived intratumoral heterogeneity (ITH) scores for preoperative prediction of high-risk subtypes in invasive lung adenocarcinoma. The integration of 2D and 3D topology-aware radiomic representations with a stacking ensemble framework is a notable strength, particularly given the demonstrated improvement over conventional radiomics (AUC 0.875 vs. 0.834) and clinicoradiological models. The use of SHAP-based interpretability and external validation via TCIA enhances methodological rigor and clinical relevance. However, several limitations reduce the overall impact. First, the manuscript does not sufficiently contextualize the proposed ITH formulation within the broader radiomics literature; the discussion of feature extraction, reproducibility, and cross-institutional variability remains limited despite known challenges in radiomics standardization. Second, while the topology-driven clustering is innovative, the sensitivity of the method to hyperparameters (e.g., fixed K=6 in k-means, sliding window size) and imaging heterogeneity is not analyzed, raising concerns regarding robustness. Third, the comparison with conventional radiomics is somewhat constrained, as advanced radiomics pipelines (e.g., harmonization techniques, deep radiomics, or hybrid DL–radiomics models) are not included. Additionally, segmentation relies on manual delineation, which may introduce observer bias and limits scalability in real-world deployment. To strengthen the manuscript, the authors should expand the methodological discussion and explicitly position their work within established radiomics frameworks. In particular, it is strongly recommended to cite and discuss "Radiomics in Medical Imaging: Methods, Applications, and Challenges", which provides a comprehensive overview of radiomics pipelines, feature stability, and clinical translation challenges. Incorporating this reference would improve the theoretical grounding and highlight how the proposed topology-based ITH scores address—or differ from—known radiomics limitations.

**Response:** We thank Reviewer #1 for the positive assessment and constructive suggestions. We have revised the manuscript to better position the proposed topology-aware ITH score within the broader radiomics literature. Specifically, we added discussion of conventional radiomics limitations, including feature instability, reproducibility concerns, validation bias, domain shift, cross-institutional variability, and clinical translation barriers. We incorporated and discussed the recommended review, *Radiomics in Medical Imaging: Methods, Applications, and Challenges*, in the Introduction/Discussion to strengthen the theoretical framing of our work.

Regarding robustness, we added an empirical analysis to justify the fixed cluster number. Candidate values of $K = 2$–8 were evaluated using the Calinski-Harabasz index, and the maximum was observed at $K = 6$, supporting the selected clustering granularity. We also clarified multicenter CT variability and explained that all images met predefined thin-section CT acquisition criteria and were processed using a uniform pipeline. We did not apply ComBat or other retrospective statistical harmonization in the primary analysis because the study aimed to evaluate model performance under real-world multicenter imaging variability and because inappropriate harmonization may remove biologically meaningful between-tumor differences. This issue has also been acknowledged as a limitation.

We agree that the comparison with conventional radiomics does not cover every advanced radiomics or deep learning pipeline. We therefore clarified that our comparison focused on a standard reproducible radiomics baseline and added this as a limitation.

For segmentation, we added an interobserver reproducibility analysis in 100 randomly selected cases. The interobserver ICC for the 3D ITH score was 0.916, supporting good robustness to manual segmentation variability. We also expanded the limitation section to discuss automated segmentation as an important future direction.

**Changes made in the revised manuscript:** We revised the Introduction and Discussion to cite and discuss the recommended radiomics review. We added S2 Fig and corresponding Methods/Results text to justify $K = 6$ using the Calinski-Harabasz index. We revised the Methods and Limitations to clarify multicenter CT heterogeneity and the rationale for not applying statistical harmonization in the primary analysis. We added a segmentation reproducibility analysis in 100 cases, reporting ICC = 0.916 for the 3D ITH score.

## Reviewer #2

### Reviewer #2 comment

> **Reviewer/editor comment:** The manuscript presents a well-structured multicenter study proposing topologically distinct intratumoral heterogeneity (ITH) scores derived from CT imaging for preoperative prediction of high-risk subtypes in invasive lung adenocarcinoma (IAC). The integration of 2D and 3D ITH metrics with clinicoradiological features and the use of a stacking ensemble framework demonstrate solid methodological rigor. The reported performance (AUC = 0.875) and external validation on TCIA data strengthen the credibility and translational relevance of the study. The inclusion of SHAP-based interpretability and ablation analysis is a notable strength, particularly the identification of the 3D ITH score as the dominant predictor ($\sim$25.5% contribution), which aligns with the objective of capturing spatial tumor heterogeneity. However, several aspects require refinement to enhance scientific depth and positioning within existing literature. First, while the study emphasizes topological characterization of heterogeneity, the conceptual linkage between radiomics-derived features and underlying biological phenotypes remains underdeveloped. The manuscript treats clustered radiomic patterns as proxies for tumor subregions, yet lacks a rigorous discussion of how these correspond to histopathological phenotypes or microenvironmental variations. This creates a gap between computational heterogeneity modeling and biological interpretability. In this context, the authors are strongly encouraged to incorporate and discuss "Phenotyping of Histology Imaging Data with Histomics", which provides a comprehensive framework for explicit phenotype-level representation, including morphology, spatial organization, and texture descriptors. Integrating insights from this work would significantly strengthen the manuscript by: (i) grounding the proposed ITH scores within a broader phenotype-centric paradigm, (ii) clarifying how clustered radiomic subregions relate to interpretable tissue phenotypes, and (iii) improving the discussion on representation-level interpretability beyond SHAP-based feature attribution. Methodologically, the reliance on k-means clustering with a fixed K=6 lacks justification and sensitivity analysis. Given that intratumoral heterogeneity is highly scale-dependent, the robustness of ITH scores to clustering granularity should be evaluated. Additionally, although the study highlights superiority over conventional radiomics, the comparison pipeline (feature filtering + LASSO) appears standard and may not reflect current best practices such as deep radiomics or self-supervised feature learning. From a clinical perspective, the manuscript would benefit from clearer discussion on deployment feasibility. Manual segmentation (ITK-SNAP) introduces observer variability and limits scalability, yet no inter-/intra-observer variability analysis is reported. Furthermore, although survival correlations are shown, causal interpretation remains limited without multivariate survival modeling controlling for confounders.

**Response:** We thank Reviewer #2 for the detailed and constructive comments. We expanded the biological and conceptual interpretation of the proposed ITH scores. Specifically, we now explain that the clustered radiomic subregions should be interpreted as CT-derived imaging phenotypes or tumor habitats that summarize local differences in attenuation, texture, and spatial organization. A higher 3D ITH score indicates more fragmented, spatially intermixed, and less dominant connected phenotypic components. Such patterns may correspond to mixed histological growth patterns, heterogeneous cellular density, stromal reaction, necrotic/fibrotic foci, or uneven invasive fronts. To avoid overinterpretation, we explicitly state that the 3D ITH score is a CT-derived spatial phenotype associated with aggressive tumor architecture rather than direct evidence of a specific molecular pathway.

We incorporated and discussed the recommended *Phenotyping of Histology Imaging Data with Histomics* article in the Discussion. Because this work focuses on histology imaging, we used it as a conceptual analogy for phenotype-centric representation rather than as direct evidence for CT imaging. This allowed us to better explain how our clustered radiomic subregions relate to morphology, texture, spatial organization, and phenotype-level interpretability.

In response to the methodological concerns, we added the Calinski-Harabasz index analysis for $K = 2$–8 and found the maximum at $K = 6$. This analysis is now presented as S2 Fig and described in Methods and Results. We also agree that advanced deep radiomics and self-supervised learning are important future directions, but they were beyond the scope of the current topology-focused framework. We clarified that the radiomics comparison was designed as a conventional reproducible baseline and acknowledged this limitation.

Regarding clinical deployment, we added a segmentation reproducibility analysis based on 100 randomly selected cases independently re-segmented by a second radiologist. The ICC for the 3D ITH score was 0.916, indicating excellent interobserver agreement. We also added future automated segmentation as an important direction to improve scalability. Finally, we agree that the TCIA survival/genomic analyses are exploratory because of the small external sample size and limited number of events. We revised the Discussion and Limitations to avoid causal interpretation and to state that larger external cohorts with multivariable survival modeling are needed.

**Changes made in the revised manuscript:** We added the histomics reference and expanded the Discussion on phenotype-centric interpretation. We added the $K = 6$ justification analysis (S2 Fig), segmentation reproducibility analysis (ICC = 0.916), discussion of deployment feasibility and automated segmentation, and a clearer limitation regarding the exploratory nature of TCIA survival/genomic analyses without multivariable confounder adjustment.

## Reviewer #3

### Reviewer #3 general comment

**Reviewer/editor comment:** This manuscript presents a multicenter machine learning framework integrating topologically distinct intratumoral heterogeneity (ITH) scores with clinicoradiological features for predicting high-risk invasive lung adenocarcinoma subtypes. The topic is clinically important, and the integration of topology-aware imaging biomarkers with ensemble learning methods is innovative. The multicenter design and inclusion of external TCIA validation strengthen the overall study. The manuscript is generally well organized and technically sound. The proposed stacking ensemble model demonstrated improved performance compared with conventional clinicoradiological and radiomics-based approaches. In particular, the finding that the 3D ITH score emerged as the dominant predictive feature is interesting and potentially clinically meaningful. However, several issues should be addressed before the manuscript can be considered for publication.

**Response:** We thank Reviewer #3 for recognizing the clinical importance, methodological novelty, multicenter design, and external validation of our study. We have addressed each major and minor comment in detail below.

### Major comment 1. Recall/sensitivity and class imbalance

**Reviewer/editor comment:** Although the stacking ensemble achieved a strong AUC, the recall/sensitivity remained relatively low. Since the stated goal is preoperative identification of aggressive MP/S tumors, sensitivity is clinically important. The authors should further discuss the clinical implications of the low recall, possible effects of class imbalance, and whether threshold optimization or cost-sensitive approaches were explored.

**Response:** We agree that sensitivity is clinically important for preoperative identification of MP/S-predominant high-risk tumors. In the previous version, threshold-dependent metrics were calculated using a default threshold, which favored specificity/precision and resulted in low recall. In the revised analysis, we recalculated threshold-dependent metrics using the optimal diagnostic threshold derived from ROC analysis. This substantially improved the balance between sensitivity and precision while retaining the threshold-independent discriminative advantage of the stacking model. The stacking ensemble achieved accuracy 0.816 (95% CI: 0.773–0.859), AUC 0.875 (95% CI: 0.830–0.920), F1-score 0.588 (95% CI: 0.468–0.708), precision 0.592 (95% CI: 0.469–0.715), and recall/sensitivity 0.584 (95% CI: 0.461–0.707). We also expanded the Discussion to emphasize that the model should be interpreted as a preoperative risk-stratification tool rather than a stand-alone rule-out test.

**Changes made in the revised manuscript:** The Statistical analysis section, Results, Discussion, and Tables 2–4 were revised. Tables 2–4 now report accuracy, AUC, F1-score, precision, and recall with 95% CIs. The Discussion now explicitly addresses the clinical implications of threshold optimization and the need to balance missed high-risk cases against unnecessary overtreatment.

**Revised Table 2.** Comparison of diagnostic performance between base machine learning models and the stacking ensemble classifier.

Table 1: *

| Models | Accuracy (95% CI) | AUC (95% CI) | F1-score (95% CI) | Precision (95% CI) | Recall (95% CI) |
|---|---|---|---|---|---|
| Stacking ensemble | 0.816 (0.773–0.859) | 0.875 (0.830–0.920) | 0.588 (0.468–0.708) | 0.592 (0.469–0.715) | 0.584 (0.461–0.707) |
| RF | 0.808 (0.765–0.851) | 0.845 (0.798–0.892) | 0.538 (0.415–0.661) | 0.542 (0.418–0.666) | 0.535 (0.411–0.659) |
| XGBoost | 0.805 (0.761–0.849) | 0.794 (0.743–0.845) | 0.515 (0.391–0.639) | 0.518 (0.393–0.643) | 0.512 (0.388–0.636) |
| LightGBM | 0.812 (0.769–0.855) | 0.828 (0.779–0.877) | 0.551 (0.428–0.674) | 0.554 (0.430–0.678) | 0.548 (0.424–0.672) |
| GBDT | 0.806 (0.762–0.850) | 0.834 (0.786–0.882) | 0.528 (0.405–0.651) | 0.531 (0.407–0.655) | 0.525 (0.401–0.649) |
| AdaBoost | 0.810 (0.767–0.853) | 0.852 (0.806–0.898) | 0.555 (0.432–0.678) | 0.558 (0.434–0.682) | 0.552 (0.428–0.676) |
| CatBoost | 0.814 (0.771–0.857) | 0.865 (0.819–0.911) | 0.575 (0.454–0.696) | 0.578 (0.455–0.701) | 0.572 (0.449–0.695) |

Abbreviations: AUC, area under the receiver operating characteristic curve; CI, confidence interval; RF, random forest.

## Major comment 2. Imaging harmonization and overfitting prevention

**Reviewer/editor comment:** Additional details regarding imaging harmonization and overfitting prevention are needed. The study includes multiple institutions and several boosting-based models, but the manuscript does not fully explain how scanner variability and protocol heterogeneity were addressed.

**Response:** We agree. We clarified that all included CT examinations were restricted to non-contrast thin-section chest CT with reconstructed slice thickness $\leq 1.5$ mm and that all analyses used a uniform preprocessing and feature-extraction pipeline, including consistent lung-window settings, tumor-mask handling, and local feature extraction parameters. We did not apply retrospective statistical harmonization such as ComBat in the primary analysis because the study aimed to evaluate model behavior under real-world multicenter acquisition variability and because inappropriate harmonization may remove biologically meaningful between-tumor differences. We acknowledged residual multicenter imaging variability as a limitation. To address overfitting, we clarified that preprocessing, feature selection, threshold determination for threshold-dependent metrics, and hyperparameter tuning were confined to the training workflow, while the internal validation and TCIA datasets were kept independent for model evaluation.

**Changes made in the revised manuscript:** The Patient population/data acquisition, Machine learning framework, Statistical analysis, and Limitations sections were revised to clarify scanner/protocol variability, the rationale for not applying ComBat in the primary analysis, and overfitting prevention procedures.

### Major comment 3. Manual segmentation and reproducibility

**Reviewer/editor comment:** The segmentation process relied on manual delineation by expert radiologists. More information regarding interobserver reproducibility or segmentation robustness would improve confidence in the proposed framework. Discussion of future automated segmentation approaches would also strengthen the translational relevance.

**Response:** We agree. We added an interobserver reproducibility analysis. In 100 randomly selected cases, a second radiologist independently re-segmented tumors while blinded to histopathological subtype and the original masks. The interobserver reproducibility of the 3D ITH score was excellent, with an ICC of 0.916. This supports the robustness of the proposed topological metric to manual segmentation variability. We also expanded the limitations to state that manual segmentation remains labor-intensive and that fully automated deep learning segmentation could improve clinical scalability.

**Changes made in the revised manuscript:** The CT lesion mask segmentation section, Results, and Limitations now report the 100-case reproducibility analysis and ICC = 0.916. Future automated segmentation has been added as a translational direction.

### Major comment 4. Biological interpretation of the 3D ITH score

**Reviewer/editor comment:** The biological interpretation of the topology-aware ITH score should be clarified further. Although mathematically well described, the clinical and biological meaning of higher 3D ITH values may not be intuitive to all readers.

**Response:** We agree and have expanded the biological interpretation in the Discussion. We now explain that a higher 3D ITH score indicates that intratumoral phenotypic clusters are more fragmented, spatially intermixed, and less dominated by a single connected component. Such a topology may reflect mixed histological growth patterns, heterogeneous cellular density, stromal reaction, necrotic/fibrotic foci, and uneven invasive fronts. We explicitly avoid overclaiming direct molecular mechanisms and interpret the 3D ITH score as a CT-derived spatial phenotype associated with aggressive tumor architecture.

**Changes made in the revised manuscript:** The Discussion now includes an expanded paragraph explaining the biological and clinical meaning of higher 3D ITH values.

### Major comment 5. Small TCIA cohort and exploratory survival/genomic analyses

**Reviewer/editor comment:** The external validation cohort from TCIA was relatively small, particularly for survival and genomic subgroup analyses. This limitation should be acknowledged more explicitly.

**Response:** We agree. We revised the Limitations to state more explicitly that the TCIA validation cohort was relatively small, especially for survival and genomic subgroup analyses. Therefore, these analyses should be interpreted as exploratory and require confirmation in larger external cohorts with multivariable survival modeling.

**Changes made in the revised manuscript:** The Limitations section now states that the TCIA survival and genomic analyses are exploratory and require validation in larger cohorts with multivariable adjustment.

### Minor comment 1. Rationale for selecting $K = 6$

**Reviewer/editor comment:** The rationale for selecting K = 6 in the clustering step should be clarified.

**Response:** We agree. We added a cluster-number selection analysis based on the Calinski-Harabasz index across $K = 2–8$. The maximum value occurred at $K = 6$, supporting the selected clustering granularity.

**Changes made in the revised manuscript:** A new S2 Fig and corresponding Methods/Results descriptions were added.

### Minor comment 2. Confidence intervals

**Reviewer/editor comment:** Confidence intervals for major performance metrics should be consistently reported.

**Response:** We agree. We added 95% confidence intervals for all major diagnostic metrics, including accuracy, AUC, F1-score, precision, and recall.

**Changes made in the revised manuscript:** Tables 2–4 were revised to include 95% confidence intervals for all performance metrics.

### Minor comment 3. Figure readability

**Reviewer/editor comment:** Several figures, particularly the SHAP and heatmap visualizations, would benefit from improved readability and larger labels.

**Response:** We agree. We improved the readability of figure files where applicable and reorganized selected figures into Supporting information to reduce the visual burden in the main manuscript. The revised figures were prepared as separate high-resolution files for submission.

**Changes made in the revised manuscript:** Selected figures were reorganized as S1–S6 Figs. Figure files will be uploaded separately according to PLOS figure requirements.

### Minor comment 4. Grammar and formatting

**Reviewer/editor comment:** Minor grammatical and formatting inconsistencies should be corrected during revision.

**Response:** We agree. We carefully revised the manuscript for grammar, terminology, statistical formatting, and consistency of abbreviations and figure/table references.

**Changes made in the revised manuscript:** The manuscript was edited for language and formatting consistency.

## Additional revisions

### True topology-aware formulas in the schematic

**Reviewer/editor comment:** No specific reviewer comment. During revision, we also reviewed the schematic formulas and corrected them to match the actual ITH-score definitions.

**Response:** We corrected the schematic formula in the revised workflow figure. The figure now uses the true topology-aware ITH-score formulas: 2D ITH $= 1 - \frac{1}{S_{\text{total}}} \sum_{i=1}^{K} \frac{S_{i,\max}}{n_i}$ and 3D ITH $= 1 - \frac{1}{V_{\text{total}}} \sum_{i=1}^{K} \frac{V_{i,\max}}{m_i}$.

**Changes made in the revised manuscript:** The workflow figure was regenerated with the correct mathematical expressions and without third-party visual elements.

### Supporting information renumbering

**Reviewer/editor comment:** No specific reviewer comment. During revision, we reorganized selected figures into Supporting information to improve manuscript readability and figure compliance.

**Response:** We reorganized selected figures as Supporting information and renumbered them from S1 Fig to S6 Fig.

**Changes made in the revised manuscript:** The revised manuscript now cites S1–S6 Figs for patient enrollment, cluster-number selection, stacking framework, diagnostic performance, comparative diagnostic performance, and meta-learner decision analysis.

We appreciate the reviewers' helpful suggestions and believe that these revisions have improved the rigor, transparency, and clinical interpretability of the manuscript.

Sincerely,

**The Authors**
