# Supplementary material for: Topologically distinct intratumoral heterogeneity scores for predicting high-risk pathological grades in invasive lung adenocarcinoma: A multicenter study across four institutions
Source: PLOS Digit Health. 2026 Sep 8;5(9):e0001646. doi: 10.1371/journal.pdig.0001646 (PMC13552742; doi:10.1371/journal.pdig.0001646)
Supplement: Peer review history

# Peer review: Decision Letter 0

**DOI:** 10.1371/journal.pdig.0001646.r001

**Authors:** Ashkan Ebadi, Fnu Neha

29 May 2026

scores for predicting high-risk pathological grades in

invasive lung adenocarcinoma: A multicenter study

Response to Reviewers Revised Manuscript with Track Changes Manuscript **Journal Requirements:**

1. In this instance it seems there may be acceptable restrictions in place that prevent the public sharing of your minimal data. However, in line with our goal of ensuring long-term data availability to all interested researchers, PLOS’ Data Policy states that authors cannot be the sole named individuals responsible for ensuring data access (http://journals.plos.org/plosone/s/data-availability#loc-acceptable-data-sharing-methods).

Data requests to a non-author institutional point of contact, such as a data access or ethics committee, helps guarantee long term stability and availability of data. Providing interested researchers with a durable point of contact ensures data will be accessible even if an author changes email addresses, institutions, or becomes unavailable to answer requests.

Before we proceed with your manuscript, please also provide non-author contact information (phone/email/hyperlink) for a data access committee, ethics committee, or other institutional body to which data requests may be sent. If no institutional body is available to respond to requests for your minimal data, please consider if there any institutional representatives who did not collaborate in the study, and are not listed as authors on the manuscript, who would be able to hold the data and respond to external requests for data access? If so, please provide their contact information (i.e., email address). Please also provide details on how you will ensure persistent or long-term data storage and availability.

2. Some material included in your submission may be copyrighted. According to PLOS’s copyright policy, authors who use figures or other material (e.g., graphics, clipart, maps) from another author or copyright holder must demonstrate or obtain permission to publish this material under the Creative Commons Attribution 4.0 International (CC BY 4.0) License used by PLOS journals. Please closely review the details of PLOS’s copyright requirements here: PLOS Licenses and Copyright. If you need to request permissions from a copyright holder, you may use PLOS's Copyright Content Permission form.

Please respond directly to this email or email the journal office and provide any known details concerning your material's license terms and permissions required for reuse, even if you have not yet obtained copyright permissions or are unsure of your material's copyright compatibility.

Potential Copyright Issues:

a. Figures 2, 3: Please confirm whether you drew the images / clip-art within the figure panels by hand. If you did not draw the images, please provide (a) a link to the source of the images or icons and their license / terms of use; or (b) written permission from the copyright holder to publish the images or icons under our CC-BY 4.0 license. Alternatively, you may replace the images with open source alternatives. See these open source resources you may use to replace images / clip-art:

- https://commons.wikimedia.org

- https://openclipart.org/

b. Figure 3 contains branding/a logo. We are not permitted to publish this under our CC-BY 4.0 license, even with permission. We ask that you please remove or replace it.

3. Please ensure that your Ethics Statement is available in its entirety at the beginning of your Methods section, under a subheading 'Ethics Statement'.

4. We ask that a manuscript source file is provided at Revision. Please upload your manuscript file as a .doc, .docx, .rtf or .tex.

5. Please upload separate figure files in .tif or .eps format. Also, remove the figures from your manuscript file but keep the legends.

For more information about figure files please see our guidelines:

https://journals.plos.org/digitalhealth/s/figures

https://journals.plos.org/digitalhealth/s/figures#loc-file-requirements

**Additional Editor Comments (if provided):** **Reviewers' Comments:**

**Comments to the Author**

1. Does this manuscript meet PLOS Digital Health’s publication criteria?

Reviewer #1: Yes

Reviewer #2: Yes

Reviewer #3: Partly

**********

2. Has the statistical analysis been performed appropriately and rigorously?

Reviewer #1: Yes

Reviewer #2: Yes

Reviewer #3: Yes

**********

3. Have the authors made all data underlying the findings in their manuscript fully available (please refer to the Data Availability Statement at the start of the manuscript PDF file)?

The PLOS Data policy

Reviewer #1: Yes

Reviewer #2: Yes

Reviewer #3: No

**********

4. Is the manuscript presented in an intelligible fashion and written in standard English?

Reviewer #1: Yes

Reviewer #2: Yes

Reviewer #3: Yes

**********

**Reviewer #1:**  The manuscript presents a well-structured multicenter study proposing topologically derived intratumoral heterogeneity (ITH) scores for preoperative prediction of high-risk subtypes in invasive lung adenocarcinoma. The integration of 2D and 3D topology-aware radiomic representations with a stacking ensemble framework is a notable strength, particularly given the demonstrated improvement over conventional radiomics (AUC 0.875 vs. 0.834) and clinicoradiological models. The use of SHAP-based interpretability and external validation via TCIA enhances methodological rigor and clinical relevance. However, several limitations reduce the overall impact. First, the manuscript does not sufficiently contextualize the proposed ITH formulation within the broader radiomics literature; the discussion of feature extraction, reproducibility, and cross-institutional variability remains limited despite known challenges in radiomics standardization. Second, while the topology-driven clustering is innovative, the sensitivity of the method to hyperparameters (e.g., fixed K=6 in k-means, sliding window size) and imaging heterogeneity is not analyzed, raising concerns regarding robustness. Third, the comparison with conventional radiomics is somewhat constrained, as advanced radiomics pipelines (e.g., harmonization techniques, deep radiomics, or hybrid DL–radiomics models) are not included. Additionally, segmentation relies on manual delineation, which may introduce observer bias and limits scalability in real-world deployment. To strengthen the manuscript, the authors should expand the methodological discussion and explicitly position their work within established radiomics frameworks. In particular, it is strongly recommended to cite and discuss “Radiomics in Medical Imaging: Methods, Applications, and Challenges”, which provides a comprehensive overview of radiomics pipelines, feature stability, and clinical translation challenges. Incorporating this reference would improve the theoretical grounding and highlight how the proposed topology-based ITH scores address—or differ from—known radiomics limitations.

**Reviewer #2:**  The manuscript presents a well-structured multicenter study proposing topologically distinct intratumoral heterogeneity (ITH) scores derived from CT imaging for preoperative prediction of high-risk subtypes in invasive lung adenocarcinoma (IAC). The integration of 2D and 3D ITH metrics with clinicoradiological features and the use of a stacking ensemble framework demonstrate solid methodological rigor. The reported performance (AUC = 0.875) and external validation on TCIA data strengthen the credibility and translational relevance of the study. The inclusion of SHAP-based interpretability and ablation analysis is a notable strength, particularly the identification of the 3D ITH score as the dominant predictor (~25.5% contribution), which aligns with the objective of capturing spatial tumor heterogeneity.

However, several aspects require refinement to enhance scientific depth and positioning within existing literature. First, while the study emphasizes topological characterization of heterogeneity, the conceptual linkage between radiomics-derived features and underlying biological phenotypes remains underdeveloped. The manuscript treats clustered radiomic patterns as proxies for tumor subregions, yet lacks a rigorous discussion of how these correspond to histopathological phenotypes or microenvironmental variations. This creates a gap between computational heterogeneity modeling and biological interpretability.

In this context, the authors are strongly encouraged to incorporate and discuss “Phenotyping of Histology Imaging Data with Histomics”, which provides a comprehensive framework for explicit phenotype-level representation, including morphology, spatial organization, and texture descriptors. Integrating insights from this work would significantly strengthen the manuscript by: (i) grounding the proposed ITH scores within a broader phenotype-centric paradigm, (ii) clarifying how clustered radiomic subregions relate to interpretable tissue phenotypes, and (iii) improving the discussion on representation-level interpretability beyond SHAP-based feature attribution.

Methodologically, the reliance on k-means clustering with a fixed K=6 lacks justification and sensitivity analysis. Given that intratumoral heterogeneity is highly scale-dependent, the robustness of ITH scores to clustering granularity should be evaluated. Additionally, although the study highlights superiority over conventional radiomics, the comparison pipeline (feature filtering + LASSO) appears standard and may not reflect current best practices such as deep radiomics or self-supervised feature learning.

From a clinical perspective, the manuscript would benefit from clearer discussion on deployment feasibility. Manual segmentation (ITK-SNAP) introduces observer variability and limits scalability, yet no inter-/intra-observer variability analysis is reported. Furthermore, although survival correlations are shown, causal interpretation remains limited without multivariate survival modeling controlling for confounders.

**Reviewer #3:**  This manuscript presents a multicenter machine learning framework integrating topologically distinct intratumoral heterogeneity (ITH) scores with clinicoradiological features for predicting high-risk invasive lung adenocarcinoma subtypes. The topic is clinically important, and the integration of topology-aware imaging biomarkers with ensemble learning methods is innovative. The multicenter design and inclusion of external TCIA validation strengthen the overall study.

The manuscript is generally well organized and technically sound. The proposed stacking ensemble model demonstrated improved performance compared with conventional clinicoradiological and radiomics-based approaches. In particular, the finding that the 3D ITH score emerged as the dominant predictive feature is interesting and potentially clinically meaningful.

However, several issues should be addressed before the manuscript can be considered for publication.

Major comments:

Although the stacking ensemble achieved a strong AUC, the recall/sensitivity remained relatively low. Since the stated goal is preoperative identification of aggressive MP/S tumors, sensitivity is clinically important. The authors should further discuss the clinical implications of the low recall, possible effects of class imbalance, and whether threshold optimization or cost-sensitive approaches were explored.

Additional details regarding imaging harmonization and overfitting prevention are needed. The study includes multiple institutions and several boosting-based models, but the manuscript does not fully explain how scanner variability and protocol heterogeneity were addressed.

The segmentation process relied on manual delineation by expert radiologists. More information regarding interobserver reproducibility or segmentation robustness would improve confidence in the proposed framework. Discussion of future automated segmentation approaches would also strengthen the translational relevance.

The biological interpretation of the topology-aware ITH score should be clarified further. Although mathematically well described, the clinical and biological meaning of higher 3D ITH values may not be intuitive to all readers.

The external validation cohort from TCIA was relatively small, particularly for survival and genomic subgroup analyses. This limitation should be acknowledged more explicitly.

Minor comments:

The rationale for selecting K = 6 in the clustering step should be clarified.

Confidence intervals for major performance metrics should be consistently reported.

Several figures, particularly the SHAP and heatmap visualizations, would benefit from improved readability and larger labels.

Minor grammatical and formatting inconsistencies should be corrected during revision.

Overall, this is a promising and clinically relevant study with meaningful methodological novelty, but further clarification and refinement are needed before publication.

**********

what does this mean?). If published, this will include your full peer review and any attached files.

**Do you want your identity to be public for this peer review?**  If you choose “no”, your identity will remain anonymous but your review may still be made public.

For information about this choice, including consent withdrawal, please see our Privacy Policy

Reviewer #1: No

Reviewer #2: No

Reviewer #3: No

**********

**Figure resubmission:**

**Reproducibility:**

To enhance the reproducibility of your results, we recommend that authors of applicable studies deposit laboratory protocols in protocols.io, where a protocol can be assigned its own identifier (DOI) such that it can be cited independently in the future. Additionally, PLOS ONE offers an option to publish peer-reviewed clinical study protocols. Read more information on sharing protocols at https://plos.org/protocols?utm_medium=editorial-email&utm_source=authorletters&utm_campaign=protocols

# Author comment: Author response to Decision Letter 1

**DOI:** 10.1371/journal.pdig.0001646.r002

9 Jun 2026

Attachment Submitted filename: Response to Reviewers.pdf

# Peer review: Decision Letter 1

**DOI:** 10.1371/journal.pdig.0001646.r003

**Authors:** Ashkan Ebadi

21 Jul 2026

Topologically distinct intratumoral heterogeneity scores for predicting high-risk pathological grades in invasive lung adenocarcinoma: A multicenter study across four institutions

PDIG-D-26-00745R1

Dear Mr Lin,

We are pleased to inform you that your manuscript 'Topologically distinct intratumoral heterogeneity scores for predicting high-risk pathological grades in invasive lung adenocarcinoma: A multicenter study across four institutions' has been provisionally accepted for publication in PLOS Digital Health.

Before your manuscript can be formally accepted you will need to complete some formatting changes, which you will receive in a follow-up email from a member of our team.

Please note that your manuscript will not be scheduled for publication until you have made the required changes, so a swift response is appreciated.

IMPORTANT: The editorial review process is now complete. PLOS will only permit corrections to spelling, formatting or significant scientific errors from this point onwards. Requests for major changes, or any which affect the scientific understanding of your work, will cause delays to the publication date of your manuscript.

If your institution or institutions have a press office, please notify them about your upcoming paper to help maximize its impact. If they'll be preparing press materials, please inform our press team as soon as possible -- no later than 48 hours after receiving the formal acceptance. Your manuscript will remain under strict press embargo until 2 pm Eastern Time on the date of publication. For more information, please contact digitalhealth@plos.org.

Thank you again for supporting Open Access publishing; we are looking forward to publishing your work in PLOS Digital Health.

Best regards,

Ashkan Ebadi

Section Editor

PLOS Digital Health

***********************************************************

**Additional Editor Comments (if provided):**

**Reviewer Comments (if any, and for reference):**

Reviewer's Responses to Questions

**Comments to the Author**

Reviewer #1: All comments have been addressed

Reviewer #3: All comments have been addressed

**********

publication criteria?

Reviewer #1: Yes

Reviewer #3: Yes

**********

3. Has the statistical analysis been performed appropriately and rigorously?

Reviewer #1: N/A

Reviewer #3: Yes

**********

4. Have the authors made all data underlying the findings in their manuscript fully available (please refer to the Data Availability Statement at the start of the manuscript PDF file)?

The PLOS Data policy

Reviewer #1: Yes

Reviewer #3: Yes

**********

5. Is the manuscript presented in an intelligible fashion and written in standard English?

Reviewer #1: Yes

Reviewer #3: Yes

**********

Reviewer #1: Author has made all the suggested changes.

Reviewer #3: I appreciate the authors' careful and thorough responses to the reviewers' comments. The revised manuscript has been substantially improved. The requested revisions regarding data availability, ethics reporting, figure compliance, and manuscript clarity have been adequately addressed. The study remains methodologically sound, clinically relevant, and well validated, and I have no further major concerns. I believe the manuscript is now suitable for publication in PLOS Digital Health.

**********

what does this mean?). If published, this will include your full peer review and any attached files.

**Do you want your identity to be public for this peer review?**  If you choose “no”, your identity will remain anonymous but your review may still be made public.

For information about this choice, including consent withdrawal, please see our Privacy Policy

Reviewer #1: No

Reviewer #3: No

**********
